# Rapid Identification of Characteristic Chemical Constituents of *Panax ginseng*, *Panax quinquefolius*, and *Panax japonicus* Using UPLC-Q-TOF/MS

**DOI:** 10.1155/2022/6463770

**Published:** 2022-02-15

**Authors:** Liu Jinbiao, Zhang Xinyue, Yang Shenshen, Wang Shuo, Liu Chengcheng, Yang Bin, Li Yubo, Cai Ting

**Affiliations:** ^1^School of Chemistry and Chemical Engineering, Tianjin University of Technology, Tianjin 300384, China; ^2^Tianjin University of Traditional Chinese Medicine, No. 10 Poyang Lake Road, West Zone, Tuanbo New City, Jinghai District, Tianjin 301617, China; ^3^Hwa Mei Hospital, University of Chinese Academy of Sciences (Ningbo No. 2 Hospital), Ningbo 315010, China; ^4^Ningbo Institute of Life and Health Industry, University of Chinese Academy of Sciences, Ningbo 315010, China

## Abstract

Saponins are the main active components in *Panax ginseng* C. A. Mey. (PG), *Panax quinquefolius* L. (PQ), and *Panax japonicus* C. A. Mey. (PJ), which belong to the genus *Panax* in the Araliaceae family. Because the chemical components in the three species are similar, they are often mixed and misused in functional foods and pharmaceuticals applications. Therefore, it is urgent to establish a method to quickly distinguish among PG, PQ, and PJ. Ultraperformance liquid chromatography quadrupole time-of-flight mass spectrometry (UPLC-Q-TOF/MS) was combined with data postprocessing to identify the main characteristic fragments (CFs) and the related neutral losses (NLs) of protopanaxadiol (PPD), protopanaxatriol (PPT), oleanolic acid (OLE), and ocotillol- (OCO-) type saponins. By comparing the mass spectral data, it was possible to rapidly classify and identify saponins in PG, PQ, and PJ. A total of twenty-three chemical components were identified in the PG samples, twenty-three components were identified in the PQ samples, and twenty-seven components were identified in the PJ samples. Among them, OCO-type saponins were characteristic of PQ and PJ. Ginsenoside Rf, which was absent from PQ, allowed for differentiation between PQ and PJ. The CFs and NLs in the mass spectra of the characteristic components of PG, PQ, and PJ allowed for the rapid classification and identification of these species. Additionally, these results provide technical support for the quality evaluation of Chinese herbal medicine and for constructing a scientific regulatory system.

## 1. Introduction

*Panax ginseng* C. A. Mey. (PG), *Panax quinquefolius* L. (PQ), and *Panax japonicus* C. A. Mey. (PJ) are three important plants of the genus *Panax* in the Araliaceae family. Based on their morphology, these plants can be divided into two groups: the first is an upright rhizome with developed fleshy roots, mainly containing dammarane- (DAM-) type tetracyclic triterpenoid saponins, such as PG, PQ, *Panax notoginseng*, and so on . The other is a developed rhizome, horizontal bamboo whip or rosary, with less fleshy roots. It mainly contains oleanolic acid (OLE) pentacyclic triterpenoid saponins, such as PJ [1]. Recent investigations have shown that the main active components of PG, PQ, and PJ are saponins, polysaccharides, phenolic acids, and alkaloids. Recent pharmacological studies have shown that saponins can delay aging, improve immunity, prevent and treat Alzheimer's disease, and regulate the nervous system. Additionally, saponins exhibit antitumor activity, along with antioxidative, antihypertensive, and antihyperglycemic properties [2–6]. Consequently, ginsenosides are widely used in food, healthcare products, cosmetics, and medicine. Although the three traditional Chinese medicinal herbs from the genus *Panax* have different pharmacological actions, indications, and clinical applications, the properties and chemical composition of these Chinese herbal species are very similar, and thus adulterated products are often passed off as genuine in the market [7–10]. For example, in order to reduce the production cost or simply via mistaken identity, PQ is added to commercial PG products [11], and narrow-leaf *Panax japonicus* and *Panax notoginseng* of the same or different families and genera are often used as adulterants intentionally or mistakenly as a substitute for genuine PJ [12]. Adulterants not only compromise the integrity of the Chinese herbal medicine market but also affect the efficacy and safety of traditional Chinese medicine. Therefore, it is urgent to establish methods for the rapid identification of the three genuses of *Panax* used in traditional Chinese medicines so as to improve the efficacy of quality evaluation and provide scientific regulation.

The ginsenosides found in PG can be divided into two groups according to their glycosidic structure: DAM-type and OLE-type. There are two types of DAM: protopanaxadiol- (PPD-) type saponins, for which the aglycone is 20(s)-PPD; these contain the most ginsenosides, including ginsenoside Rb_1_, Rb_2_, Rb_3_, Rc, Rd, Rg_3_, and Rh_2_, and protopanaxatriol- (PPT-) type saponins, for which the aglycone is 20(s)-PPT, including ginsenoside Re, Rf, Rg_1_, and Rh_1_. The aglycone of OLE-type ginsenosides, such as ginsenoside Ro, is oleanolic acid [13]. Compared to PG, PQ and PJ not only contain PPD-, PPT-, and OLE-type saponins but also contain ocotillol- (OCO-) type saponins, such as pseudoginsenoside F_11_ and pseudoginsenoside RT_4_ [14, 15]. In addition, ginsenoside Rf has not been found in PQ [16]. The types of saponins, similar to the structures of their parental nucleus, are rich and complex. Therefore, it is necessary to develop a rapid method for the qualitative analysis of saponins that allows for the accurate classification and identification of different traditional Chinese medicines from the genus *Panax*.

**Figure 1:**
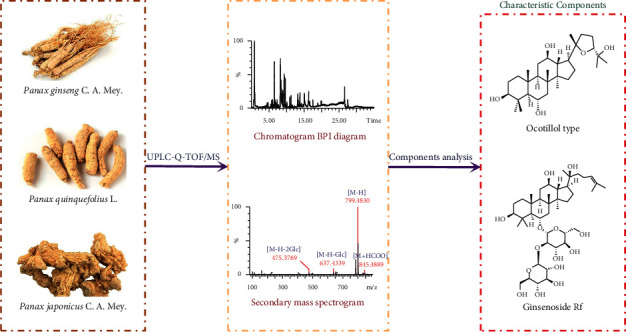
The rapid identification strategy of three traditional Chinese medicines in the genus *Panax*.

In this study, an accurate, rapid, and sensitive ultraperformance liquid chromatography quadrupole tandem time-of-flight mass spectrometry (UPLC-Q-TOF/MS) technique combined with data postprocessing is established (Figure 1). First, the characteristic fragments (CFs) and neutral losses (NLs) of various saponins are summarized. Based on the quasi-molecular ions and the fragment ions provided by high-resolution mass spectrometry, the chromatographic retention time, and related literature data, the saponin profiles of PG, PQ, and PJ are identified in order to realize accurate distinction between the three. This study aims to explore the medicinal basis of the three traditional Chinese medicinal herbs from the genus *Panax* and provide basic information for establishing a comprehensive system for evaluating the quality of medicinal materials. Simultaneously, this approach can provide technical support for constructing a scientifically based regulatory system.

## 2. Materials and Methods

### 2.1. Materials, Reagents, and Instruments

Nine batches of representative medicinal materials were collected or purchased from Jilin, the main area producing PG and PQ, and from different areas producing PJ. The detailed sample information is presented in Table 1. High-performance liquid chromatography-grade acetonitrile was provided by Oceanpak (Sweden), high-performance liquid chromatography-grade formic acid was provided by Thermo Fisher (USA), and distilled water was purchased from Watsons Food and Beverage Company (China). A Waters Acquity (Waters, USA) UPLC instrument and a Xevo G2 (Waters, USA) Q-TOF/MS system were used in this study.

### 2.2. Sample Preparation

The Chinese medicinal herbs PG-1, PQ-1, and PJ-1 were, respectively, crushed, and 0.2 g of the powdered PG-1, PQ-1, and PJ-1 was placed into three separate test tubes, soaked in 10 mL of 70% ethanol, and ultrasonically extracted for 50 min. After extraction, each tube was cooled and centrifuged for 10 min. The supernatant was subsequently filtered through a 0.22 *μ*m microporous membrane and analyzed by UPLC-Q-TOF/MS.

### 2.3. UPLC and MS Conditions

UPLC conditions were as follows: a Waters Acquity UPLC BEH C18 column (2.1 mm× 100 mm, 1.7 *µ*m) was used as the chromatographic column. The column temperature was set at 40°C, the flow rate was 0.3 mL/min, the injection volume was 5 *µ*L, the mobile phase was composed of 0.1% formic acid aqueous solution (A) and acetonitrile (B), and the chromatographic separation was carried out by gradient elution, where the gradient sequence was as follows: 0–2 min, 5–10% B; 2–6 min, 10–30% B; 6–10 min, 30–50% B; 10–15 min, 50–80% B; 15–20 min, 80–100% B; 20–25 min, 100% B; 25–30 min, 100–5% B; and 30–35 min, 5% B.

TOF-MS conditions were as follows: mass spectrometry was performed using a Waters G2 Q-TOF mass spectrometer, equipped with a negative mode electrospray ionization source. The capillary voltage was −2.4 kV, the cone voltage was 40 V, the source temperature was 120°C, the desolvation temperature was 400°C, the desolvation gas was 800 L/h, and the cone gas was 50 L/h, using leucine enkephalin (*m/z* 554.2615) as an external reference. In order to ensure the accuracy of the data acquisition, the full-scan data in the range of 100–1500 Da were obtained.

### 2.4. Method Establishment

The main pharmacological constituents of PG, PQ, and PJ are saponins. Therefore, to accurately distinguish the three traditional Chinese medicines, it was necessary to classify and identify the saponins. However, the use of conventional methods to determine the composition of saponins is complicated and time-consuming because of their large molecular weight and similar core structure. In collision-induced MS, compounds with the same or similar parent nuclear skeletons usually fracture similarly, and this technique is used to establish fragmentation patterns. CFs are molecular compounds with the same or similar parent core structures. When exposed to the energy impact of MS, they can fragment into ions, from which the cleavage type and material can be easily inferred. CFs can be used to help to rapidly classify the target materials. In addition, molecular ions can lose neutral radicals or molecules in MS, as shown by the difference between the mass/load ratio and the molecular ion peak and the product ion peaks, respectively. These lost free-radicals or molecules are known as NLs, which aid the screening and identification of substances [17–22]. Therefore, we present the MS fragmentation of PG, PQ, and PJ and summarize their CFs and common NLs, which are based on the different core structures (DAM-, OLE-, and OCO-types). First, the different CFs were used to preliminarily classify the unknown components. The various saponins were identified by combined analysis of their molecular ions, retention time, and the fragmentation pattern of the unknown components, along with their fracture processes, which were estimated using common NLs. Based on the types of saponins in the samples, the three traditional Chinese medicinal herbs could be identified quickly and accurately.

## 3. Results and Discussion

**Figure 2:**
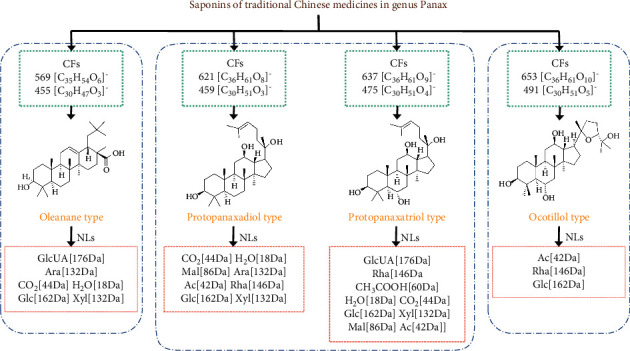
Characteristic fragments and neutral losses of different types of saponins in genus *Panax*. Ac: acetyl; Mal: malonyl; Glc: glucose residue; Ara: arabinose residue; Rha: rhamnose residue; Xyl: xylose residue; GlcUA: glucuronic acid.

**Figure 3:**
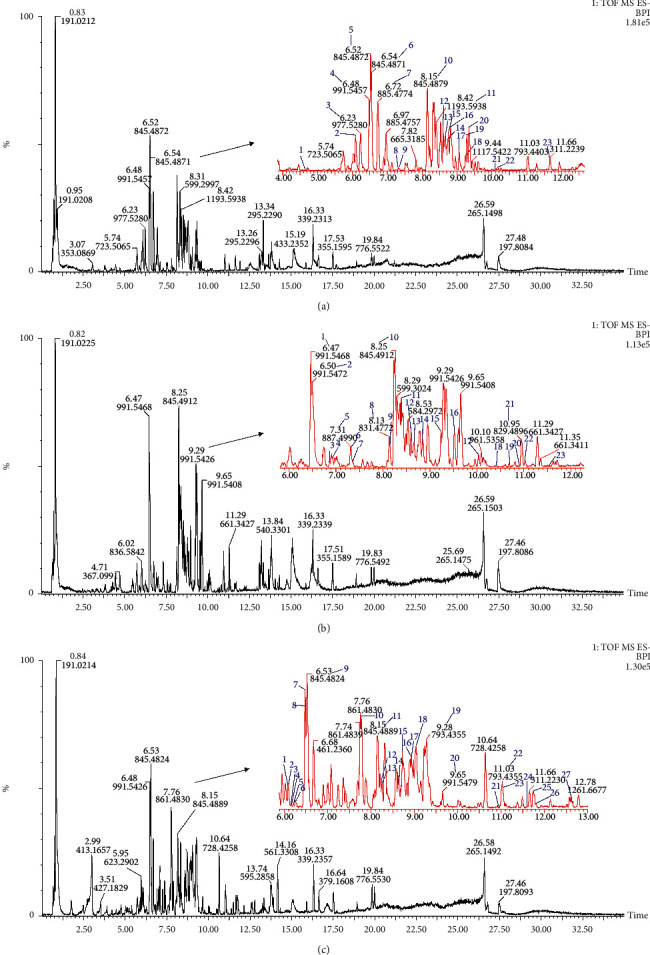
Chromatogram BPI diagram of PG, PJ, and PQ under negative ions (a) PG, (b) PQ, and (c) PJ.

Based on the summarized CF and NL data, PG, PQ, and PJ were analyzed. Twenty-three chemical constituents were identified for the PG samples, which included 10 PPD saponins, 11 PPT saponins, and 2 OLE saponins. A total of twenty-three components was identified from PQ, which included 12 PPD saponins, 4 PPT saponins, 3 OLE saponins, and 4 OCO saponins. A total of twenty-seven components was identified in the PJ samples, which included 7 PPD saponins, 6 PPT saponins, 11 OLE saponins, and 3 OCO saponins. The CFs and NLs of the different types of saponins are shown in Figure 2. The total ion chromatograms of the PG, PQ, and PJ extracts in negative ion mode are shown in Figure 3, and their compositions are shown in Tables 2–4.

### 3.1. Analysis of Dammarane-Type Saponins by MS

#### 3.1.1. PPD-Type Saponins

PPD-type ginsenosides, such as ginsenosides Rb, Rb_2_, Rc, and Rg_3_, are saponins in the genus *Panax*. In 1966, Shibata et al. isolated ginsenediol from the root of ginseng for the first time and reported its chemical properties and structure [35]. Considering the structural types of PPD and the mass spectral information in the literature, it was found that two CFs were produced, with signals at *m/z* 621 [C_36_H_61_O_8_]^−^ and *m/z* 459 [C_30_H_51_O_3_]^−^. At the same time, the product ions observed in the MS^2^ profiles of the PPD-type saponins generally resulted in the following NLs: CO_2_ (44 Da), H_2_O (18 Da), Mal (86 Da), Ara (132 Da), Glc (162 Da), Xyl (132 Da), Ac (42 Da), and Rha (146 Da). Therefore, based on the CFs and NLs, it was possible to identify the compounds and infer their fracture processes.

**Figure 4:**
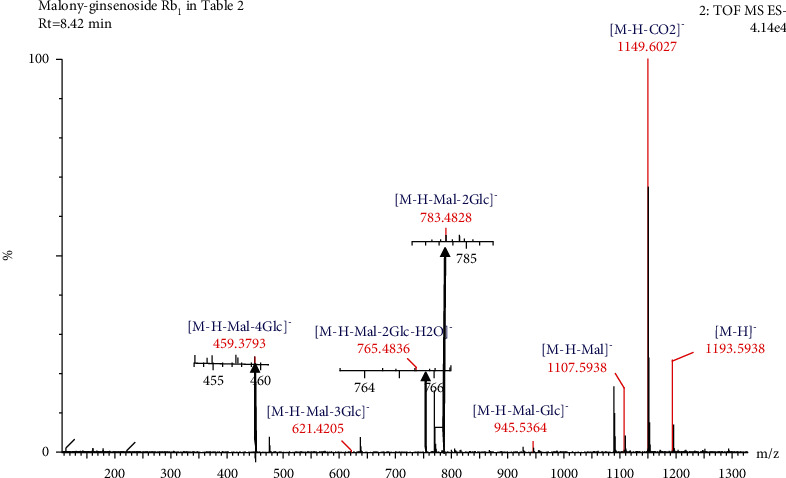
Secondary mass spectrogram of malonyl-ginsenoside Rb_1_.

**Figure 5:**
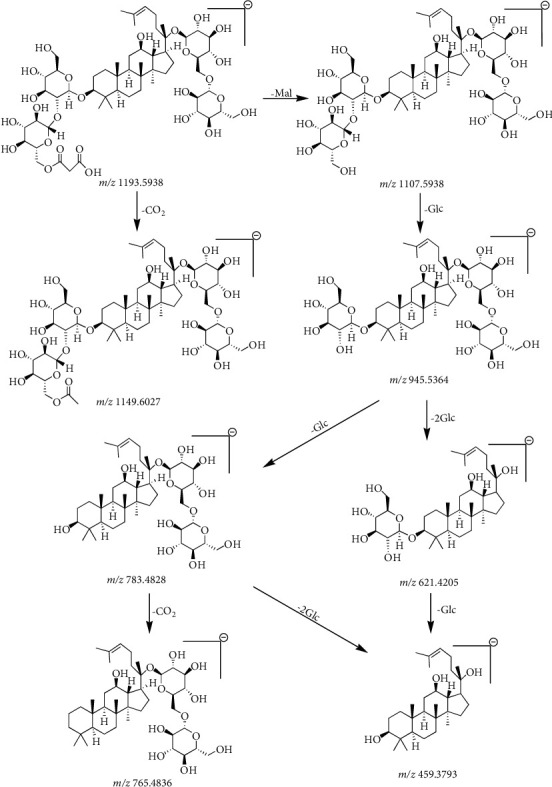
The fragmentation pathway of malonyl-ginsenoside Rb_1_.

Compound 11 (Table 2) had a retention time of 8.42 min and a molecular formula of C_57_H_94_O_26_. In the negative ion mode, compound 11 produced a precursor ion at *m/z* 1193.5938 [M-H]^−^ and seven fragment ion peaks at *m/z* 1149.6027, 1107.5938, 945.5364, 783.4828, 765.4836, 621.4205, and 459.3793. Based on the CF ions at *m/z* 621.4205 and 459.3793, compound 11 in Table 2 could be preliminarily identified as a PPD-type saponin. The product ion at *m/z* 1149.6027 was produced by the removal of a CO_2_ molecule (44 Da) from the precursor ion. The product ion at *m/z* 1107.5938 was produced by the malonyl group (86 Da) of the precursor ion. The *m/z* 945.5364 product ion was produced by the neutral loss of malonyl and a part of the glucose residue (162 Da) from the precursor ion. When the product ions at *m/z* 945.5364 continued to lose glucose residues, product ions with *m/z* 783.4828 [M-H-Mal-2Glc]^−^, *m/z* 621.4205 [M-H-Mal-3Glc]^−^, and *m/z* 459.3793[M-H-Mal-4Glc]^−^ were formed. When the product ion with a peak at *m/z* 783.4828 lost one H_2_O molecule (18 Da), the product ion at *m/z* 765.4836 [M-H-Mal-2Glc-H_2_O]^−^ was formed. Therefore, compound 11 (Table 2) was identified as malonyl-ginsenoside Rb_1_ from its molecular ion and secondary mass spectral fracture pattern [24, 30]. The cleavage pathway of malonyl-ginsenoside Rb_1_ in negative ion mode is shown in Figures 4 and 5.

#### 3.1.2. PPT-Type Saponins

Thus far, PPT-type saponins, such as ginsenoside Re and ginsenoside Rg_1_, have been found in ginseng plants. Notably, PQ did not contain ginsenoside Rf. The CFs of these saponins occurred at *m/z* 637 [C_36_H_61_O_9_]^−^ and *m/z* 475 [C_30_H_51_O_4_]^−^ and include GlcUA (176 Da), Rha (146 Da), CH_3_COOH (60 Da), H_2_O (18 Da), CO_2_ (44 Da), Glc (162 Da), Xyl (132 Da), Mal (86 Da), and Ac (42 Da). Therefore, the PPT saponins could be preliminarily identified by these product ions. This was further supported by the different NLs, which were caused by the breaking of different substituents at the C-6 and C-20 positions.

**Figure 6:**
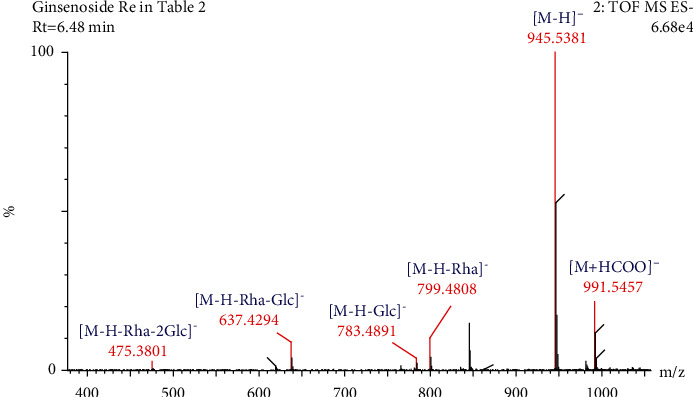
Secondary mass spectrogram of ginsenoside Re.

**Figure 7:**
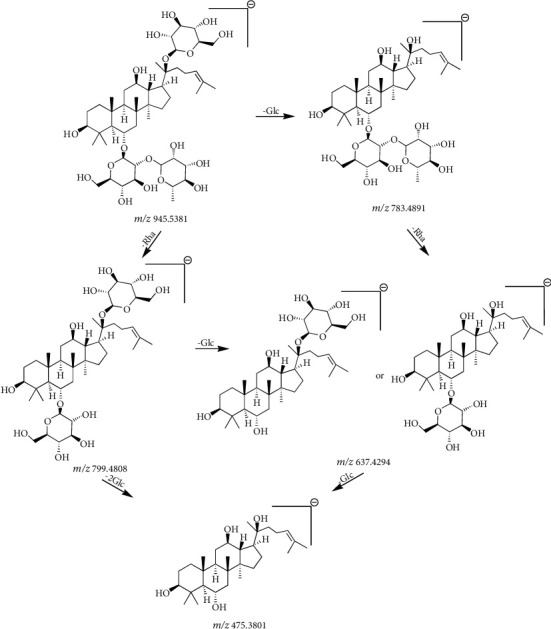
The fragmentation pathway of ginsenoside Re.

Compound 4 (Table 2) had a retention time of 6.48 min and a molecular formula of C_48_H_82_O_18_. In negative ion mode, this compound had a molecular ion peak at *m/z* 991.5457 [M + HCOO]^−^ and product ion peaks at *m/z* 945.5381 [M-H]^−^, 799.4808 [M-H-Rha]^−^, 783.4891 [M-H-Glc]^−^, 637.4294 [M-H-Glc-Rha]^−^, and 475.3801 [M-H-2Glc-Rha]^−^. Based on the CFs at *m/z* 637.4294 and 475.3801, the compound was identified as a PPT-type saponin. Based on the molecular ion, fragments, and reference information, compound 4 was identified as ginsenoside Re [24–27, 30]. The cleavage pathway was as follows: the molecular ion at *m/z* 945.5381 [M-H]^−^ lost one glucose residue (162 Da) at C-20 to generate the product ion peak at *m/z* 783.4891 [M-H-Glc]^−^, while the molecular ion at *m/z* 945.5381 [M-H]^−^ lost one rhamnose residue (146 Da) at C-6 to generate the product ion at *m/z* 799.4808 [M-H-Rha]^−^. When the molecular ions simultaneously lost a glucose and rhamnose residue (162 Da + 146 Da), a product ion peak was produced at *m/z* 637.4294 [M-H-Glc-Rha]^−^. The fragment ion at *m/z* 475.3801 [M-H-2Glc-Rha]^−^ was produced when the product ion at *m/z* 783.4891 lost a glucose and rhamnose residue (162 Da + 146 Da) simultaneously. The fragmentation pathway of ginsenoside Re in negative-ion mode is shown in Figures 6 and 7.

**Figure 8:**
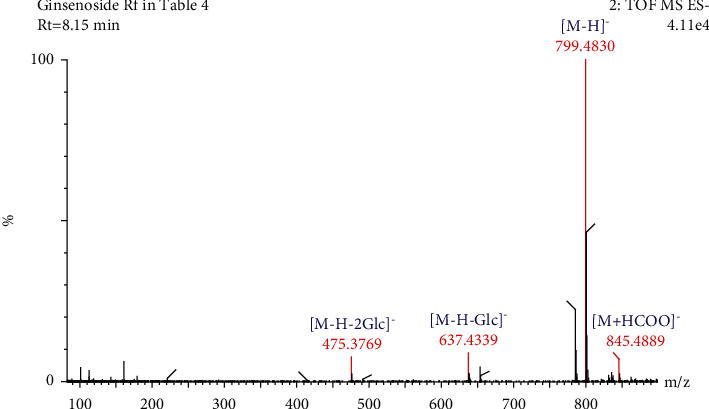
Secondary mass spectrogram of ginsenoside Rf.

**Figure 9:**
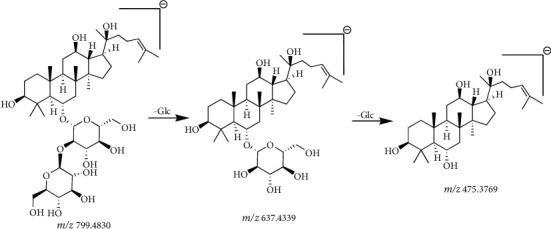
The fragmentation pathway of ginsenoside Rf.

Compound 11 (Table 4) had a retention time of 8.15 min and molecular formula of C_42_H_72_O_14_. In negative ion mode, compound 11 produced a precursor ion at *m/z* 845.4889 [M + HCOO]^−^ and three fragment ion peaks at *m/z* 799.4830, 637.4339, and 475.3769. Based on the product ion peaks at *m/z* 637.4339 and 475.3769, compound 11 was preliminarily considered to be a PPT-type saponin. When this observation was further combined with the retention time, along with the molecular ion and fragment information, compound 11 (Table 4) was identified as ginsenoside Rf [31, 32]. The cleavage pathway of ginsenoside Rf was as follows: the product ion peak at *m/z* 637.4339 was generated by the loss of one glucose residue (162 Da) from the molecular ion at *m/z* 799.4830. The fragment ion peak at *m/z* 475.3769 was generated when the product ion at *m/z* 637.4339 lost one glucose residue (162 Da). The fragmentation information and process for ginsenoside Rf are shown in Figures 8 and 9.

### 3.2. Analysis of OLE-Type Saponins by MS

Pentacyclic triterpenoid saponins of the OLE-type are characteristic components of ginseng. There are differences in the species and availability of different ginseng plants [36]. The CFs of the OLE-type saponins occurred at *m/z* 569 [C_35_H_54_O_6_]^−^ and 455 [C_30_H_47_O_3_]^−^, and the common neutral losses corresponded to GlcUA (176 Da), Ara (132 Da), CO_2_ (44 Da), H_2_O (18 Da), Glc (162 Da), and Xyl (132 Da). Therefore, the OLE-type saponins could be quickly identified and described using the CF information and the retention times of the fractured C-3 and C-28 ester bases.

**Figure 10:**
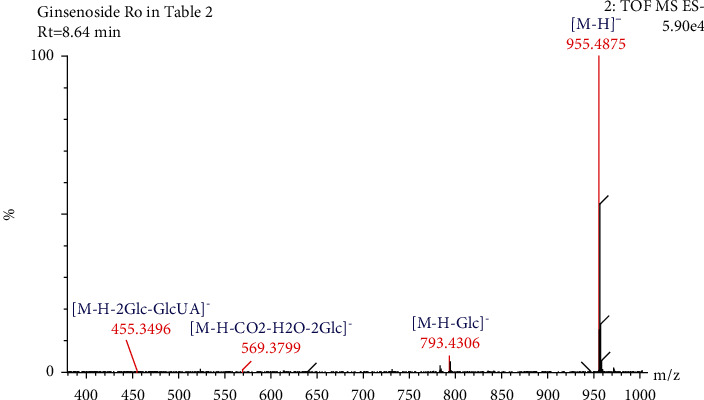
Secondary mass spectrogram of ginsenoside Ro.

**Figure 11:**
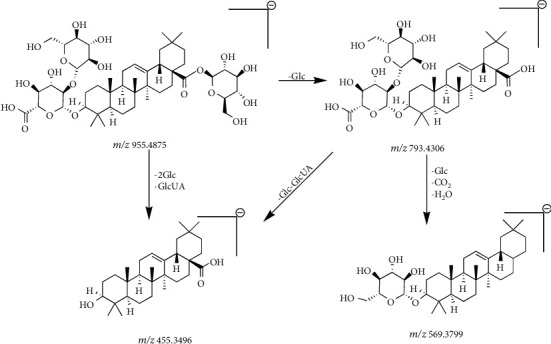
The fragmentation pathway of ginsenoside Ro.

Compound 13 (Table 2) had a retention time of 8.64 min and a molecular formula of C_48_H_76_O_19_. In negative ion mode, compound 13 was detected by the molecular ion peak at *m/z* 955.4875 [M-H]^−^ and product ion peaks at *m/z* 793.4306 [M-H-Glc]^−^, 569.3799 [M-H-CO_2_-H_2_O-2Glc]^−^, and 455.3496 [M-H-2Glc-GlcUA]^−^. Based on the CFs at *m/z* 569.3799 and 455.3496, the compound was preliminarily determined to be an OLE-type saponin. Combining the mass spectrometry and the remaining fragment ion information (Table 2), compound 13 was identified as ginsenoside Ro [27–29]. The fragmentation of ginsenoside Ro occurred as follows: when the molecular ion at *m/z* 955.4875 [M-H]^−^ lost one molecular glucose residue (162 Da), fragment ion peaks were generated at *m/z* 793.4306, when the molecular ions lost one CO_2_ molecule (44 Da), one H_2_O (18 Da), and two molecular glucose residues (162 Da + 162 Da), the product ion peak at *m/z* 569.3799 was produced. When the ions at *m/z* 955.4875 [M-H]^−^ lost two molecular glucose residues (162 Da + 162 Da) and one glucuronic acid molecule (176 Da), a resultant ion peak appeared at *m/z* 455.3496. The fragmentation of ginsenoside Ro is shown in Figures 10 and 11.

### 3.3. Analysis of OCO-Type Saponins by Mass Spectrometry

A furan ring was introduced into the C-20 and C-24 positions of the dammarane skeleton through a connection with oxygen, which resulted in the formation of an OCO-type saponin [15]. Studies have shown that ginseng does not contain such saponins, and as a characteristic feature, the types and contents of OCO-type saponins in PQ and PJ are also different. The results showed that the ions at *m/z* 653 [C_36_H_61_O_9_]^−^ and *m/z* 491 [C_30_H_51_O_5_]^−^ were CFs associated with OCO-type saponins. Ac (42 Da), Rha (146 Da), and Glc (162 Da) were the common NL fragments. From this information, the general cleavage behavior of OCO-type saponins from PQ and PJ could be identified and proposed.

**Figure 12:**
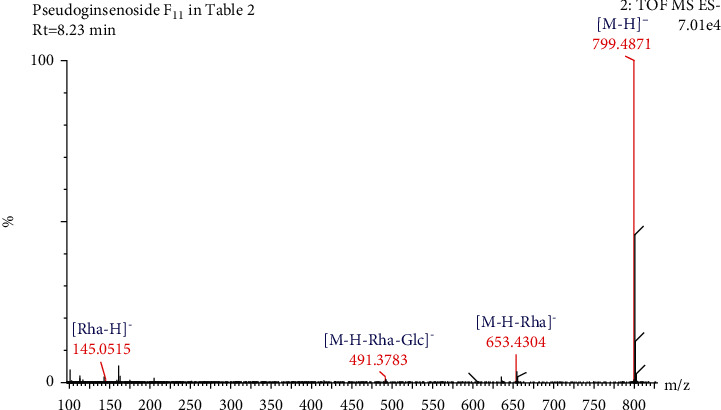
Secondary mass spectrogram of pseudoginsenoside F_11_.

**Figure 13:**
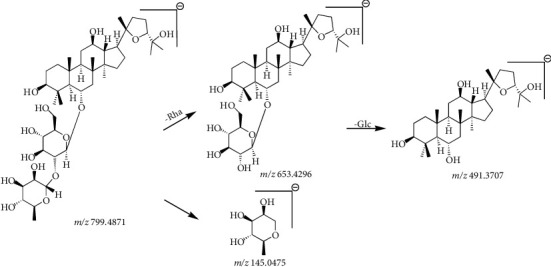
The fragmentation pathway of pseudoginsenoside F_11_.

Compound 10 in Table 3, with a retention time of 8.25 min and a molecular formula of C_42_H_72_O_14_, generated molecular ion peaks at *m/z* 845.4912 [M + HCOO]^−^ and fragment ion peaks at *m/z* 799.4871, 653.4296, 491.3707, and 145.0475 in negative ion mode. Based on the CF ion peaks at *m/z* 653.4296 and *m/z* 491.3707, the compound was identified as an OCO-type saponin. When this observation was combined with the literature data and molecular weight, the compound was identified as pseudoginsenoside F_11_ [33, 34]. The cleavage pathway is as follows: when the molecular ion at *m/z* 799.4871 [M-H]^−^ lost one molecular rhamnose residue (146 Da), the product ion at *m/z* 653.4296 [M-H-Rha]^−^ was produced. Subsequently, the fragment ion peak at *m/z* 491.3707 [M-H-Rha-Glc]^−^ was produced by the loss of one molecular rhamnose residue and one molecular glucose residue (146 Da + 162 Da). In negative ion mode, the product ion peak at *m/z* 145.0475 [Rha-H]^−^ was examined for free rhamnose residues. The cleavage pathway of pseudoginsenoside F_11_ is shown in Figures 12 and 13.

### 3.4. Analysis of Differences in Saponins

**Figure 14:**
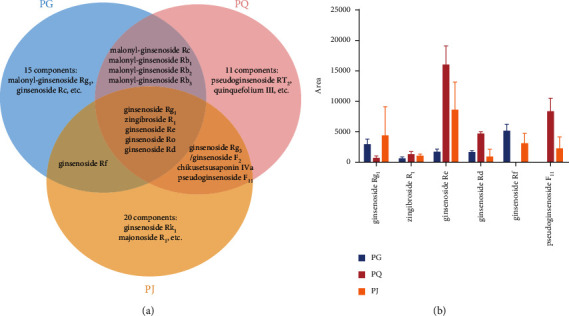
(a) Venn diagram of the distribution of saponins among PG, PQ, and PJ. (b) Contents of six components in PG, PQ, and PJ.

From identification of the chemical components, the characteristic absence of ginsenoside Rf in PQ was noted; on this premise, PQ can be differentiated. OCO saponins were not found in PG, which could be useful in identifying PG and PJ. Based on the information in Tables 2–4, the distribution of saponins among PG, PQ, and PJ are shown in the Venn diagram in Figure 14(a). The results show that ginsenoside Rg_1_, zingibroside R_1_, ginsenoside Re, ginsenoside Ro, and ginsenoside Rd were the common components of PG, PQ, and PJ. Based on this information, the ginsenoside content in PG, PQ, and PJ was preliminarily analyzed. The main ginsenosides (Rg_1_, Re, Rb_1_, Rc, and Rd) generally account for more than 70% of the total content of ginsenosides in PQ [37]. The common components, the characteristic components of the three traditional Chinese medicines, along with ginsenoside Rg_1_, zingibroside R_1_, ginsenoside Re, ginsenoside Rd, ginsenoside Rf, and OCO-type saponins (taking pseudoginsenoside F_11_ as an example), were selected for comparison [38, 39]. The contents of these six components in the nine batches of medicinal materials were analyzed (Figure 14(b)). These results show that the common chemical components in PG, PQ, and PJ were present in significantly different contents and that characteristic components only existed in specific medicinal materials. Considering that the differences in the saponins in PG, PQ, and PJ were preliminarily analyzed, future studies on the three kinds of medicinal materials from the genus *Panax* are needed. Nonetheless, the results have provided a foundation for the quantitative study of PG, PQ, and PJ and for screening the pharmacological components.

## 4. Conclusions

In this study, fragment ions associated with the chemical constituents in PG, PQ, and PJ were studied. Additionally, mass spectra fragmentation rules for the DAM-type (including PPD- and PPT-type), OLE-type, and OCO-type saponins were presented. The chemical constituents and different saponins from PG, PQ, and PJ were analyzed using UPLC-Q-TOF/MS. With the aid of the fragmentation rules for various components, 23 chemical components were identified in PG, 23 chemical components were identified in PQ, and 27 chemical components were identified in PJ. Among them, PG did not contain OCO-type saponins; thus, it was distinguishable from PQ and PJ. Additionally, ginsenoside Rf, a characteristic component, was not found in PQ, which provides a basis for differentiating between PQ and PJ. Through rapid classification and identification of the components, we differentiated among three types of traditional Chinese medicinal herbs from the genus *Panax*. This study provides a foundation for pharmacodynamic research and the development of MS in the identification of traditional Chinese medicine. Thus, this study presents a guaranteed approach for the determination of chemical components, along with the development and application of ginseng in traditional Chinese medicine.

## Figures and Tables

**Table 1:** Detailed information of the tested PG, PQ, and PJ samples.

Sample number	Source	Identity
PG-1	Jilin province, China	*Panax ginseng* C. A. Mey.
PG-2	Jilin province, China	*Panax ginseng* C. A. Mey.
PG-3	Jilin province, China	*Panax ginseng* C. A. Mey.
PQ-1	Jilin province, China	*Panax quinquefolius* L.
PQ-2	Jilin province, China	*Panax quinquefolius* L.
PQ-3	Jilin province, China	*Panax quinquefolius* L.
PJ-1	Anhui province, China	*Panax japonicus* C. A. Mey.
PJ-2	Sichuan province, China	*Panax japonicus* C. A. Mey.
PJ-3	Yunnan province, China	*Panax japonicus* C. A. Mey.

**Table 2:** Cracking information of chemical components in PG under negative ion mode.

No.	Identity	Formula	Rt	Theoretical value	Actual value	Ppm	Main MS/MS fragments detected	Saponin type	Ref.
1	Ginsenoside Re_5_	C_42_H_72_O_15_	4.65	861.4848 [M + HCOO]^−^	861.4824 [M + HCOO]^−^	−2.79	415.0735 [M-H-GlcUA-Rha-CH_3_COOH-H_2_O]^−^	PPT	[23]

2	20-O-glucosylginsenoside Rf	C_48_H_82_O_19_	6.09	1007.5427 [M + HCOO]^−^	1007.5408 [M + HCOO]^−^	−1.89	961.5369 [M-H]^−^ 799.4875 [M-H-Glc]^−^ 637.4326 [M-H-2Glc]^−^ 475.3742 [M-H-3Glc]^−^	PPT	[24]

3	Notoginsenoside R_1_	C_47_H_80_O_18_	6.23	977.5321 [M + HCOO]^−^	977.5280 [M + HCOO]^−^	−4.19	931.5211 [M-H]^−^ 799.4812 [M-H-Xyl]^−^ 637.4296 [M-H-Xyl-Glc]^−^ 475.3794 [M-H-Xyl-2Glc]^−^	PPT	[24]

4	Ginsenoside Re	C_48_H_82_O_18_	6.48	991.5478 [M + HCOO]^−^	991.5457 [M + HCOO]^−^	−2.12	945.5381 [M-H]^−^ 799.4808 [M-H-Rha]^−^ 783.4891 [M-H-Glc]^−^ 637.4294 [M-H-Glc-Rha]^−^ 475.3801 [M-H-2Glc-Rha]^−^	PPT	[24–27]

5	Ginsenoside Rg_1_	C_42_H_72_O_14_	6.52	845.4899 [M + HCOO]^−^	845.4871 [M + HCOO]^−^	−3.31	799.4819 [M-H]^−^ 637.4293 [M-H-Glc]^−^ 475.3802 [M-H-2Glc]^−^	PPT	[27, 28]

6	Ginsenoside Rg_7_	C_42_H_72_O_14_	6.54	845.4899 [M + HCOO]^−^	845.4870 [M + HCOO]^−^	−3.43	799.4816 [M-H]^−^ 637.4293 [M-H-Glc]^−^ 475.3775 [M-H-2Glc]^−^	PPT	[24, 27]

7	Malonyl-ginsenoside Rg_1_	C_45_H_74_O_17_	6.72	885.4848 [M-H]^−^	885.4771 [M-H]^−^	−8.70	841.4857 [M-H-CO_2_]^−^ 799.4765 [M-H-Mal]^−^ 781.4659 [M-H-Mal-H_2_O]^−^ 637.4263 [M-H-Mal-Glc]^−^ 619.4142 [M-H-Ma-H_2_O-Glc]^−^ 475.3755[M-H-Mal-2Glc]^−^	PPT	[27–29]

8	Yesanchinoside D (6'-O-acetyl-ginsenoside Rg_1_)	C_44_H_74_O_15_	7.31	887.5004 [M + HCOO]^−^	887.4955 [M + HCOO]^−^	−5.52	841.4865 [M-H]^−^ 781.4639 [M-CH_3_COOH]^−^	PPT	[24]

9	Notoginsenoside R_2_	C_41_H_70_O_13_	7.35	815.4793 [M + HCOO]^−^	815.4787 [M + HCOO]^−^	−0.74	769.4833 [M-H]^−^ 637.4329 [M-H-Xyl]^−^ 475.3798 [M-H-Xyl-Glc]^−^	PPT	[27, 30]

10	Ginsenoside Rf	C_42_H_72_O_14_	8.15	845.4899 [M + HCOO]^−^	845.4879 [M + HCOO]^−^	−2.37	799.4833 [M-H]^−^ 781.4752 [M-H-H_2_O]^−^ 637.4337 [M-H-Glc]^−^ 475.3816 [M-H-2Glc]^−^	PPT	[24]

11	Malonyl-ginsenoside Rb_1_	C_57_H_94_O_26_	8.42	1193.5955 [M-H]^−^	1193.5938 [M-H]^−^	−1.42	1149.6027 [M-H-CO_2_]^−^ 1107.5938 [M-H-Mal]^−^ 945.5364 [M-H-Mal-Glc]^−^ 783.4828 [M-H-Mal-2Glc]^−^ 765.4836 [M-H-Mal-2Glc-H_2_O]^−^ 621.4205 [M-H-Mal-3Glc]^−^ 459.3793 [M-H-Mal-4Glc]^−^	PPD	[24, 27, 30]

12	Malonyl-ginsenoside Rc	C_56_H_92_O_25_	8.60	1163.5849 [M-H]^−^	1163.5798 [M-H]^−^	−4.38	1119.5902 [M-H-CO_2_]− 1077.5809 [M-H-Mal]− 945.5629 [M-H-Mal-Xyl]− 783.4856 [M-H-Mal-Xyl-Glc]− 621.4377 [M-H-Mal-Xyl-2Glc]− 459.3702 [M-H-Mal- Xyl-3Glc]−	PPD	[24, 26, 27, 30]

13	Ginsenoside Ro	C_48_H_76_O_19_	8.64	955.4903 [M-H]^−^	955.4875 [M-H]^−^	−2.93	955.4875 [M-H]^−^ 793.4306 [M-H-Glc]^−^ 569.3799 [M-H-CO_2_-H_2_O-2Glc]^−^ 455.3496 [M-H-2Glc-GlcUA]^−^	OLE	[24, 27]

14	Ginsenoside Rc	C_53_H_90_O_22_	8.75	1123.5900 [M + HCOO]^−^	1123.5856 [M + HCOO]^−^	−3.92	1077.5808 [M-H]^−^ 945.5377 [M-H-Xyl]^−^ 783.4871 [M-H-Xyl-Glc]^−^ 621.4434 [M-H-Xyl-2Glc]^−^ 459.3853 [M-H-Xyl-3Glc]^−^	PPD	[24, 27, 30]

15	Ginsenoside Rb_2_/ginsenoside Rb_3_	C_53_H_90_O_22_	8.76	1123.5900 [M + HCOO]^−^	1123.5859 [M + HCOO]^−^	−3.65	1077.5814 [M-H]^−^ 945.5422 [M-H-Xyl]^−^ 783.4926 [M-H-Xyl-Glc]^−^ 621.4468 [M-H-Xyl-2Glc]^−^ 459.3786 [M-H-Xyl-3Glc]^−^	PPD	[24, 25, 27]

16	Malonyl-ginsenoside Rb_2_	C_56_H_92_O_25_	8.81	1163.5849 [M-H]^−^	1163.5817 [M-H]^−^	−2.75	1119.5912 [M-H-CO_2_]− 1077.5797 [M-H-Mal]− 945.5309 [M-H-Mal-Xyl]− 783.4660 [M-H-Mal-Xyl-Glc]− 621.4481 [M-H-Mal-Xyl-2Glc]− 459.3897 [M-H-Mal-Xyl-3Glc]−	PPD	[24, 30]

17	Malonyl-ginsenoside Rb_3_	C_56_H_92_O_25_	9.06	1163.5849 [M-H]^−^	1163.5876 [M-H]^−^	2.32	1077.5974 [M-H-Mal]^−^ 945.5316 [M-H-Mal-Xyl]^−^ 783.5035 [M-H-Mal-Xyl-Glc]^−^ 621.4245 [M-H-Mal-Xyl-2Glc]^−^ 459.3838 [M-H-Mal-Xyl-3Glc]^−^	PPD	[24]

18	Zingibroside R_1_	C_42_H_66_O_14_	9.24	793.4374 [M-H]^−^	793.4355 [M-H]^−^	−2.39	631.38266 [M-H-Glc]^−^ 569.3931 [M-H-Glc-CO_2_-H_2_O]^−^ 455.3629 [M-H-Glc-GlcUA]^−^	OLE	[27]

19	Ginsenoside Rd	C_48_H_82_O_18_	9.29	991.5478 [M + HCOO]^−^	991.5463 [M + HCOO]^−^	−1.51	945.5413 [M-H]^−^ 783.4880 [M-H-Glc]^−^ 621.4258 [M-H-2Glc]^−^ 459.3817 [M-H-3Glc]^−^ 161.0483 [Glc-H]^−^	PPD	[24, 26, 27]

20	Malonyl-ginsenoside Re	C_51_H_84_O_21_	9.35	1031.5427 [M-H]^−^	1031.5428 [M-H]^−^	0.10	1031.5460 [M-H]^−^ 987.5533 [M-H-CO_2_]^−^ 945.5430 [M-H-Mal]^−^ 783.4849 [M-H-Mal-Glc]^−^ 637.4373 [M-H-Mal-Rha-Glc]^−^ 475.3859 [M-H-Mal-Rha-2Glc]^−^	PPT	[24, 27]

21	Notoginsenoside Fe/vina-ginsenoside R_16_	C_47_H_80_O_17_	10.09	961.5372 [M + HCOO]^−^	961.5469 [M + HCOO]^−^	10.09	915.5186 [M-H]^−^ 783.5407 [M-H-Xyl]^−^ 753.5070 [M-H-Glc]^−^ 621.4315 [M-H-Xyl-Glc]^−^ 459.3875 [M-H-Xyl-2Glc]^−^	PPD	[27]

22	Malonyl-notoginsenoside Fe	C_50_H_82_O_20_	10.14	1001.5321 [M-H]^−^	1001.5320 [M-H]^−^	−0.10	783.4836 [M-H-Xyl-Mal]^−^ 459.3748 [M-H-Xyl-Mal-2Glc]^−^	PPD	[27]

23	Ginsenoside F_2_/ginsenoside Rg_3_	C_42_H_72_O_13_	11.62	829.4949 [M + HCOO]^−^	829.4916 [M + HCOO]^−^	−3.98	783.4875 [M-H]^−^ 621.4391 [M-H-Glc]^−^ 459.3772 [M-H-2Glc]^−^	PPD	[27, 31, 32]

**Table 3:** Cracking information of chemical components in PQ under negative ion mode.

No.	Identity	Formula	Rt	Theoretical value	Actual value	Ppm	Main MS/MS fragments detected	Saponin type	Ref.
1	Ginsenoside Re	C_48_H_82_O_18_	6.47	991.5478 [M + HCOO]^−^	991.5468 [M + HCOO]^−^	−1.01	945.5403 [M-H]^−^ 799.4841 [M-H-Rha]^−^ 783.4899 [M-H-Glc]^−^ 637.4333 [M-H-Glc-Rha]^−^ 475.3792 [M-H-2Glc-Rha]^−^	PPT	[28]

2	Gypenoside X VII	C_48_H_82_O_18_	6.50	991.5478 [M + HCOO]^−^	991.5472 [M + HCOO]^−^	−0.61	945.5408 [M-H]^−^ 783.4871 [M-H-Glc]^−^ 621.4116 [M-H-2Glc]^−^ 459.7427 [M-H-3Glc]^−^ 161.0451 [Glc-H]^−^	PPD	[28]

3	Malonyl-ginsenoside Rc	C_56_H_92_O_25_	6.87	1209.5904 [M + HCOO]^−^	1209.5897 [M + HCOO]^−^	−0.58	1119.4801 [M-H-CO2]− 1077.7046 [M-H-Mal]− 1077.7046 [M-H-Mal]− 783.4866 [M-H-Mal-Xyl-Glc]−	PPD	[28]

4	Ginsenoside Rg_1_	C_42_H_72_O_14_	6.93	845.4899 [M + HCOO]^−^	845.4886 [M + HCOO]^−^	−1.54	799.4836 [M-H]^−^ 637.4357 [M-H-Glc]^−^ 475.3795 [M-H-2Glc]^−^	PPT	[28]

5	Acetyl-ginsenoside Rg_1_	C_44_H_74_O_15_	7.31	887.5004 [M + HCOO]^−^	887.4990 [M + HCOO]^−^	−1.58	841.4931 [M-H]^−^ 799.4761 [M-H-Ac]^−^ 781.4734 [M-H-Ac-H_2_O]^−^ 679.4437 [M-H-Glc]^−^ 637.4263 [M-H-Ac-Glc]^−^ 619.4208 [M-H-Ac-Glc-H_2_O]^−^ 475.3736 [M-H-Ac-2Glc]^−^	PPT	[28, 31, 32]

6	Vina-ginsenoside R_1_	C_44_H_74_O_15_	7.32	887.5004 [M + HCOO]^−^	887.4999 [M + HCOO]^−^	−0.56	799.4686 [M-H-Ac]^−^ 653.3508 [M-H-Ac-Rha]^−^ 491.3585 [M-H-Ac-Rha-Glc]^−^	OCO	[28]

7	Pseudoginsenoside RC_1_	C_50_H_84_O_19_	7.35	1033.5583 [M + HCOO]^−^	1033.5575 [M + HCOO]^−^	−0.77	987.5534 [M-H]^−^ 945.5455 [M-H-Ac]^−^ 783.4812 [M-H-Ac-Glc]^−^ 621.4262 [M-H-Ac-2Glc]^−^ 459.1374 [M-H-Ac-Glc]^−^ 161.0461 [Glc-H]^−^	PPD	[28]

8	Pseudoginsenoside RT_2_	C_41_H_70_O_14_	8.13	831.4742 [M + HCOO]^−^	831.4772 [M + HCOO]^−^	3.61	785.4709 [M-H]^−^ 653.4288 [M-H-Xyl]^−^	OCO	[28]

9	Majonoside R_2_	C_41_H_70_O_14_	8.14	831.4742 [M + HCOO]^−^	831.4769 [M + HCOO]^−^	3.25	785.4690 [M-H]^−^ 653.4274 [M-H-Xyl]^−^	OCO	[28]

10	Pseudoginsenoside F_11_	C_42_H_72_O_14_	8.25	845.4899 [M + HCOO]^−^	845.4912 [M + HCOO]^−^	1.54	799.4871 [M-H]^−^ 653.4296 [M-H-Rha]^−^ 491.3707 [M-H-Rha-Glc]^−^ 145.0475 [Rha-H]^−^	OCO	[28, 33, 34]

11	Malonyl-ginsenoside Rb_1_	C_57_H_94_O_26_	8.39	1193.5955 [M-H]^−^	1193.5978 [M-H]^−^	1.93	1149.6088 [M-H-CO_2_]^−^ 1107.5940 [M-H-Mal]^−^ 945.5502 [M-H-Mal-Glc]^−^ 783.4980 [M-H-Mal-2Glc]^−^ 621.4172 [M-H-Mal-3Glc]^−^ 459.3224 [M-H-Mal-4Glc]^−^	PPD	[28]

12	Malonyl-ginsenoside Rb_2_	C_56_H_92_O_25_	8.60	1163.5849 [M-H]^−^	1163.5861 [M-H]^−^	1.03	1119.4962 [M-H-CO_2_]− 1077.5817 [M-H-Mal]− 945.5414 [M-H-Mal-Xyl]− 783.4878 [M-H-Mal-Xyl-Glc]− 621.4401 [M-H-Mal-Xyl-2Glc]− 459.2655 [M-H-Mal-Xyl-3Glc]−	PPD	[28]

13	Ginsenoside Ro	C_48_H_76_O_19_	8.65	955.4903 [M-H]^−^	955.4913 [M-H]^−^	1.05	955.4924 [M-H]^−^ 793.4407 [M-H-Glc]^−^ 569.3823 [M-H-CO_2_-H_2_O-2Glc]^−^ 455.3648 [M-H-2Glc-GlcUA]^−^	OLE	[28]

14	Malonyl-ginsenoside Rb_3_	C_56_H_92_O_25_	8.85	1163.5849 [M-H]^−^	1163.5825 [M-H]^−^	−2.06	1077.5806 [M-H-Mal]− 945.5403 [M-H-Mal-Xyl]− 783.4883 [M-H-Mal-Xyl-Glc]− 621.4357 [M-H-Mal-Xyl-2Glc]− 459.3861 [M-H-Mal-Xyl-3Glc]−	PPD	[28]

15	Zingibroside R_1_	C_42_H_66_O_14_	9.25	793.4374 [M-H]^−^	793.4337 [M-H]^−^	−4.66	793.4332 [M-H]^−^ 631.3823 [M-H-Glc]^−^ 569.3790 [M-H-Glc-CO_2_-H_2_O]^−^ 455.3538 [M-H-Glc-GlcUA]^−^	OLE	[28]

16	Ginsenoside Rd	C_48_H_82_O_18_	9.52	991.5478 [M + HCOO]^−^	991.5460 [M + HCOO]^−^	−1.82	945.5386 [M-H]^−^ 783.4875 [M-H-Glc]^−^ 621.4283 [M-H-2Glc]^−^ 459.3574 [M-H-3Glc]^−^	PPD	[28]

17	Quinquefolium III	C_50_H_84_O_19_	10.04	1033.5583 [M + HCOO]^−^	1033.5500 [M + HCOO]^−^	−8.03	945.5333 [M-H-Ac]^−^ 783.4835 [M-H-Ac-Glc]^−^ 621.4166 [M-H-Ac-2Glc]^−^ 459.3737 [M-H-Ac-3Glc]^−^ 161.0451 [Glc-H]^−^	PPD	[28]

18	Malonyl-ginsenoside Rd	C_50_H_84_O_19_	10.41	1033.5583 [M + HCOO]^−^	1033.5500 [M + HCOO]^−^	−8.03	987.5585 [M-H]− 945.5641 [M-H-Ac]− 783.4999 [M-H-Ac-Glc]− 621.4255 [M-H-Ac-2Glc]−	PPD	[28]

19	Quinquefolium I	C_52_H_86_O_19_	10.70	1059.5740 [M + HCOO]^−^	1059.5701 [M + HCOO]^−^	−3.68	945.5345 [M-H-C_4_H_4_O]^−^ 783.4911 [M-H-C_4_H_4_O-Glc]^−^ 621.4280 [M-H-C_4_H_4_O-2Glc]^−^ 161.0440 [Glc-H]^−^	PPD	[28, 29]

20	Ginsenoside Rg_5_/ginsenoside Rg_6_/ginsenoside Rk_1_/ginsenoside Rg_4_	C_42_H_70_O_12_	10.91	811.4844 [M + HCOO]^−^	811.4832 [M + HCOO]^−^	−1.48	765.4765 [M-H]^−^ 619.4148 [M-H-Rha]^−^ 603.2623 [M-H-Glc]^−^ 457.2162 [M-H-Rha-Glc]^−^ 161.0476 [Glc-H]^−^	PPD	[28]

21	Ginsenoside Rg_2_	C_42_H_72_O_13_	10.95	829.4949 [M + HCOO]^−^	829.4896 [M + HCOO]^−^	−6.39	783.4891 [M-H]^−^ 621.4318 [M-H-Glc]^−^ 459.3699 [M-H-2Glc]^−^	PPT	[28]

22	Chikusetsusaponin IVa	C_42_H_66_O_14_	11.02	793.4374 [M-H]^−^	793.4336 [M-H]^−^	−4.79	793.4336 [M-H]− 631.3883 [M-H-Glc]− 569.3820 [M-H-Glc-CO_2_-H_2_O]− 455.3252 [M-H-Glc-GlcUA]−	OLE	[28]

23	Ginsenoside Rg_3_/ginsenoside F_2_	C_42_H_72_O_13_	11.62	829.4949 [M + HCOO]^−^	829.4918 [M + HCOO]^−^	−3.74	783.4839 [M-H]^−^ 637.6862 [M-H-Rha]^−^	PPD	[28]

**Table 4:** Cracking information of chemical components in PJ under negative ion mode.

No.	Identity	Formula	Rt	Theoretical value	Actual value	Ppm	Main MS/MS fragments detected	Saponin type	Ref.
1	Notoginsenoside N/M/R_6_/R_3_/20-glc-ginsenoside-Rf	C_48_H_82_O_19_	6.08	1007.5427 [M + HCOO]^−^	1007.5358 [M + HCOO]^−^	−6.85	799.4779 [M-H-Glc]^−^ 637.4254 [M-H-2Glc]^−^	PPT	[31]

2	Ginsenoside Re_1_/Re_2_/Re_3_	C_48_H_82_O_19_	6.10	1007.5427 [M + HCOO]^−^	1007.5357 [M + HCOO]^−^	−6.95	799.4781 [M-H-Glc]^−^ 637.4270 [M-H-2Glc]^−^	PPT	[31, 32]

3	Vina-ginsenoside R_7_	C_53_H_90_O_22_	6.13	1123.5900 [M + HCOO]^−^	1123.5768 [M + HCOO]^−^	−1.75	1077.5750 [M-H]^−^ 945.5393 [M-H-Xyl]^−^ 621.2886 [M-H-Xyl-2Glc]^−^	PPD	[31, 32]

4	Ginsenoside Rb_3_/ginsenoside Rc	C_53_H_90_O_22_	6.14	1123.5900 [M + HCOO]^−^	1123.5845 [M + HCOO]^−^	−4.90	945.5501 [M-H-Xyl/Ara]^−^ 783.4982 [M-H-Xyl/Ara-Glc]^−^ 765.4770 [M-H-Xyl/Ara-Glc-H_2_O]^−^ 621.3179 [M-H-Xyl/Ara-2Glc]^−^	PPD	[31]

5	Notoginsenoside R_1_/ginsenoside Re_4_/quinquenoside L_17_	C_47_H_80_O_18_	6.23	977.5321 [M + HCOO]^−^	977.5490 [M + HCOO]^−^	17.29	931.5217 [M-H]^−^ 799.4762 [M-H-Xyl]^−^ 637.4242 [M-H-Xyl-Glc]^−^ 475.3714 [M-H-Xyl-2Glc]^−^	PPT	[31]

6	Yesanchinoside B	C_48_H_82_O_20_	6.23	977.5321 [M-H]^−^	977.5203 [M-H]^−^	−12.07	977.5415 [M-H]^−^ 653.3294 [M-H-2Glc]^−^	OCO	[31, 32]

7	Ginsenoside Re	C_48_H_82_O_18_	6.48	991.5478 [M + HCOO]^−^	991.5399 [M + HCOO]^−^	−7.97	945.5347 [M-H]^−^ 799.4775 [M-H-Rha]^−^ 783.4836 [M-H-Glc]^−^ 637.4283 [M-H-Rha-Glc]^−^ 475.3784 [M-H-2Glc-Rha]^−^	PPT	[31, 32]

8	Notoginsenoside K	C_48_H_82_O_18_	6.50	991.5478 [M + HCOO]^−^	991.5387 [M + HCOO]^−^	−9.18	945.5326 [M-H]^−^ 783.4836 [M-H-Glc]^−^	PPD	[31, 32]

9	Ginsenoside Rg_1_	C_42_H_72_O_14_	6.53	845.4899 [M + HCOO]^−^	845.4824 [M + HCOO]^−^	−8.87	799.4767 [M-H]^−^ 637.4272 [M-H-Glc]^−^ 475.3758 [M-H-2Glc]^−^	PPT	[31, 32]

10	Majonoside R_1_	C_42_H_72_O_14_	7.77	861.4848 [M + HCOO]^−^	861.4969 [M + HCOO]^−^	14.05	815.4773 [M-H]^−^ 653.4257 [M-H-Glc]^−^ 491.3711 [M-H-2Glc]^−^	OCO	[31, 32]

11	Ginsenoside Rf	C_42_H_72_O_14_	8.15	845.4899 [M + HCOO]^−^	845.4889 [M + HCOO]^−^	−1.18	799.4830 [M-H]^−^ 637.4339 [M-H-Glc]^−^ 475.3769 [M-H-2Glc]^−^	PPT	[31, 32]

12	Tuberoside A	C_48_H_76_O_19_	8.23	955.4903 [M-H]^−^	955.4896 [M-H]^−^	−0.73	793.4327 [M-H-Glc]^−^ 569.3922 [M-H-CO_2_-H_2_O-2Glc]^−^	OLE	[31, 32]

13	Pseudoginsenoside F_11_	C_42_H_72_O_14_	8.24	845.4899 [M + HCOO]^−^	845.4893 [M + HCOO]^−^	−0.71	799.4825 [M-H]^−^ 653.4245 [M-H-Rha]^−^	OCO	[32]

14	Ginsenoside Ro	C_48_H_76_O_19_	8.66	955.4903 [M-H]^−^	955.4905 [M-H]^−^	0.21	793.4402 [M-H-Glc]^−^ 613.3748 [M-H-H_2_O-2Glc]^−^ 569.3944 [M-H-2Glc-H_2_O-CO_2_]^−^ 455.3560 [M-H-2Glc-GlcUA]^−^	OLE	[31, 32]

15	Hemsgiganoside B	C_48_H_76_O_19_	8.75	955.4903 [M-H]^−^	955.4906 [M-H]^−^	0.31	793.4359 [M-H-Glc]^−^ 569.3929 [M-H-2Glc-H_2_O-CO_2_]^−^	OLE	[31, 32]

16	Stipuleanoside R_1_/chikusetsusaponin Ib	C_47_H_74_O_18_	8.92	925.4797 [M-H]^−^	925.4800 [M-H]^−^	0.32	763.3628 [M-H-Glc]^−^ 569.3837 [M-H-Glc-Ara-H_2_O-CO_2_]^−^	OLE	[31, 32]

17	Pseudoginsenoside RT_1_	C_47_H_74_O_18_	8.94	925.4797 [M-H]^−^	925.4786 [M-H]^−^	−1.19	763.4263 [M-H-Glc]^−^ 613.3727 [M-H-Glc-Xyl-H_2_O]^−^ 569.3864 [M-H-Glc-Xyl-H_2_O-CO_2_]^−^	OLE	[31, 32]

18	Chikusetsusaponin IV	C_47_H_74_O_18_	9.04	925.4797 [M-H]^−^	925.4793 [M-H]^−^	−0.43	613.3701 [M-H-Glc-Ara-H_2_O]^−^ 569.3853 [M-H-Glc-Ara-H_2_O-CO_2_]^−^	OLE	[31, 32]

19	Zingibroside R_1_	C_42_H_66_O_14_	9.28	793.4374 [M-H]^−^	793.4355 [M-H]^−^	−2.39	793.4355 [M-H]− 631.3818 [M-H-Glc]− 569.3826 [M-H-Glc-CO_2_-H_2_O]− 455.3538 [M-H-Glc-GlcUA]−	OLE	[31, 32]

20	Ginsenoside Rd	C_48_H_82_O_18_	9.65	991.5478 [M + HCOO]^−^	991.5479 [M + HCOO]^−^	0.10	945.5435 [M-H]^−^ 783.4866 [M-H-Glc]^−^ 621.4390 [M-H-2Glc]^−^ 459.3862 [M-H-3Glc]^−^ 161.0467 [Glc-H]^−^	PPD	[31, 32]

21	Ginsenoside Rg_3_/ginsenoside F_2_	C_42_H_72_O_13_	10.95	829.4949 [M + HCOO]^−^	829.4946 [M + HCOO]^−^	−0.36	783.4850 [M-H]^−^ 621.4301 [M-H-Glc]^−^ 459.3812 [M-H-2Glc]^−^	PPD	[31, 32]

22	Chikusetsusaponin IVa	C_42_H_66_O_14_	11.03	793.4374 [M-H]^−^	793.4355 [M-H]^−^	−2.39	793.4348 [M-H]^−^ 631.3970 [M-H-Glc]^−^ 569.3805 [M-H-Glc-CO_2_-H_2_O]^−^	OLE	[31, 32]

23	Cynarasaponin C	C_42_H_66_O_14_	11.05	793.4374 [M-H]^−^	793.4331 [M-H]^−^	−5.42	793.4328 [M-H]^−^ 631.3818 [M-H-Glc]^−^ 569.3826 [M-H-Glc-CO_2_-H_2_O]^−^	OLE	[31, 32]

24	Ginsenoside Rg_5_	C_42_H_70_O_12_	11.61	811.4844 [M + HCOO]^−^	811.4778 [M + HCOO]^−^	−8.13	765.4724 [M-H]^−^ 603.4313 [M-H-Glc]^−^	PPD	[31, 32]

25	Pseudoginsenoside Rp_1_	C_41_H_64_O_13_	11.77	763.4269 [M-H]^−^	763.4222 [M-H]^−^	−6.16	613.3658 [M-H-Xyl-H_2_O]− 569.3800 [M-H-Xyl-H_2_O-CO_2_]−	OLE	[31, 32]

26	Ginsenoside Rk_1_	C_42_H_70_O_12_	11.81	811.4844 [M + HCOO]^−^	811.4800 [M + HCOO]^−^	−5.42	765.4821 [M-H]^−^ 603.4160 [M-H-Glc]^−^ 161.0470 [Glc-H]^−^	PPD	[31, 32]

27	Oleanolic acid-28-O-*β*-D-glucopyranose(PJS-1)	C_36_H_58_O_8_	12.62	663.4108 [M + HCOO]^−^	663.4067 [M + HCOO]^−^	−6.18	617.4132 [M-H]^−^ 455.3546 [M-H-Glc]^−^	OLE	[31, 32]

Ac: acetyl; Mal: malonyl; Glc: glucose residue; Ara: arabinose residue; Rha: rhamnose residue; Xyl: xylose residue; GlcUA: glucuronic acid; PPD: protopanaxadiol type; PPT: protopanaxatriol type; OLE: oleanane type; OCO: ocotillol type.

## Data Availability

No data were used to support this study.

## References

[1] Zhao H., He Y., Yuan D., Zhang C. (2010). Research advances on *Panax japonicas* and its approximation varieties in Tujia nationality. *Agric Sci Technol*.

[2] Lou T., Huang Q., Su H., Zhao D., Li X. (2021). Targeting sirtuin 1 signaling pathway by ginsenosides. *Journal of Ethnopharmacology*.

[3] Zare-Zardini H., Alemi A., Taheri-Kafrani A. (2020). Assessment of a new ginsenoside Rh2 Nanoniosomal formulation for enhanced antitumor efficacy on prostate cancer: an in vitro study. *Drug Design, Development and Therapy*.

[4] Irfan M., Kwak Y. S., Han C. K., Hyun S. H., Rhee M. H. (2020). Adaptogenic effects of *Panax ginseng* on modulation of cardiovascular functions. *J Ginseng Res*.

[5] Liu H., Liu Y., Zheng W. (2014). Extraction and separation and physiological activity of the ginsenoside from ginseng stem and leaf. *Journal of Jilin Institute of Chemical Technology*.

[6] Wang Y., Pan J. Y., Xiao X. Y., Lin R. C., Cheng Y. Y. (2006). Simultaneous determination of ginsenosides in *Panax ginseng* with different growth ages using high-performance liquid chromatography-mass spectrometry. *Phytochemical Analysis*.

[7] Chen Y., Zhao Z., Chen H., Yi T., Qin M., Liang Z. (2015). Chemical differentiation and quality evaluation of commercial Asian and American ginsengs based on a UHPLC-QTOF/MS/MS metabolomics approach. *Phytochemical Analysis*.

[8] Ichim M. C., de Boer H. J. (2020). A review of authenticity and authentication of commercial ginseng herbal medicines and food supplements. *Frontiers in Pharmacology*.

[9] Nguyen V. B., Park H. S., Lee S. C., Lee J., Park J. Y., Yang T. J. (2017). Authentication markers for five major *Panax* species developed via comparative analysis of complete chloroplast genome sequences. *Journal of Agricultural and Food Chemistry*.

[10] Cui S., Wu J., Wang J., Wang X. (2017). Discrimination of American ginseng and Asian ginseng using electronic nose and gas chromatography-mass spectrometry coupled with chemometrics. *J Ginseng Res*.

[11] Wu W. R., Cheng C. S., Cheng Q. Q. (2020). Novel SNP markers on ginsenosides biosynthesis functional gene for authentication of ginseng herbs and commercial products. *Chinese Journal of Natural Medicines*.

[12] Chen J., Yang L., Li R., Zhang J., Hu M., Liu Y. (2018). Identification of *Panax japonicus* and its related species or adulterants using ITS2 sequence. *Chinese Traditional and Herbal Drugs*.

[13] Liu H., Lu X., Hu Y., Fan X. (2020). Chemical constituents of *Panax ginseng* and *Panax notoginseng* explain why they differ in therapeutic efficacy. *Pharmacological Research*.

[14] Yang W. Z., Hu Y., Wu W. Y., Ye M., Guo D. A. (2014). Saponins in the genus *Panax* L. (Araliaceae): a systematic review of their chemical diversity. *Phytochemistry*.

[15] Liu J., Xu Y., Yang J. (2017). Discovery, semisynthesis, biological activities, and metabolism of ocotillol-type saponins. *J Ginseng Res*.

[16] Yang W., Qiao X., Li K. (2016). Identification and differentiation of *Panax ginseng, Panax quinquefolium,* and *Panax notoginseng* by monitoring multiple diagnostic chemical markers. *Acta Pharmaceutica Sinica B*.

[17] Yang S., Tian M., Yuan L. (2016). Analysis of *E. rutaecarpa* alkaloids constituents in vitro and in vivo by UPLC-Q-TOF-MS combined with diagnostic fragment. *J Anal Methods Chem*.

[18] Shan L., Wu Y., Yuan L., Zhang Y., Xu Y., Li Y. (2017). Rapid screening of chemical constituents in *Rhizoma anemarrhenae* by UPLC-Q-TOF/MS combined with data postprocessing techniques. *Evid Based Complement Alternat Med*.

[19] Shan L., Yang N., Zhao Y., Sheng X., Yang S., Li Y. (2018). A rapid classification and identification method applied to the analysis of glycosides in *Bupleuri radix* and liquorice by ultra-high performance liquid chromatography coupled with quadrupole time-of-flight mass spectrometry. *Journal of Separation Science*.

[20] Yang N., Dong Y. Q., Wu M. F., Li S. Z., Yu H. X., Yang S. S. (2020). Establishing a rapid classification and identification method for the major triterpenoids of *Alisma orientale*. *Phytochemical Analysis*.

[21] Li N., Xie L., Yang N. (2021). Rapid classification and identification of chemical constituents in *Epimedium koreanum* Nakai by UPLC-Q-TOF-MS combined with data post-processing techniques. *Phytochemical Analysis*.

[22] Yang S., Zhang X., Dong Y., Sun G., Jiang A., Li Y. (2021). Cleavage rules of mass spectrometry fragments and rapid identification of chemical components of *Radix paeoniae* Alba using UHPLC-Q-TOF-MS. *Phytochemical Analysis*.

[23] Wang H. P., Yang X. B., Yang X. W. (2013). Ginsenjilinol, a new protopanaxatriol-type saponin with inhibitory activity on LPS-activated NO production in macrophage RAW 264.7 cells from the roots and rhizomes of *Panax ginseng*. *Journal of Asian Natural Products Research*.

[24] Yang Y., Yang Y., Qiu H. (2021). Localization of constituents for determining the age and parts of ginseng through ultraperfomance liquid chromatography quadrupole/time of flight-mass spectrometry combined with desorption electrospray ionization mass spectrometry imaging. *Journal of Pharmacy Biomedicine Analytical*.

[25] Liu J., Chen P., Chen L., Wang Q., Wu H., Xu R. (2016). Analysis on chemical components of *Shenqi guishao* decoction by UPLC-Q-TOF/MS. *Chin J Pharm Anal*.

[26] Jin G., Sun J., Zhang H. (2009). Identifying ginsenosides in ginseng whitening cream using electrospray ionization tandem mass spectrometry. *Modernization of traditional Chinese medicine and materia medical-world science and technology*.

[27] Guo Y. L., Wang Y., Zhao Y. L. (2020). Chemical comparison of white ginseng before and after extrusion by UHPLC-Q-Orbitrap-MS/MS and multivariate statistical analysis. *J Anal Methods Chem*.

[28] Huang X., Liu Y., Zhang Y. (2019). Multicomponent assessment and ginsenoside conversions of *Panax quinquefolium* L. roots before and after steaming by HPLC-MSn. *J Ginseng Res*.

[29] Li L., Tan L., Wang C. (2021). Identification of chemical constituents of American ginseng fruit pedicels by ultraperformance liquid chromatography quadrupole time-of-flight mass spectrometry. *Chinese Journal of Applied Chemistry*.

[30] Zhao L., Jiao C., Li H. (2018). Chemical transformation of protopanaxadiol type ginsenoside Rb_1_, Rb_2_ and Rc analyzed by RRLC-Q-TOF-MS. *Chemical Journal of Chinese Universities*.

[31] Chen J., Tan M., Zou L. (2019). Saponins in *Panacis japonici* rhizoma as analyzed by UFLC-Triple TOF MS/MS. *Food Science*.

[32] Chen J., Tan M., Zou L. (2019). Qualitative and quantitative analysis of the saponins in *Panacis japonici* rhizoma using ultra-fast liquid chromatography coupled with triple quadrupole-time of flight tandem mass spectrometry and ultra-fast liquid chromatography coupled with triple quadrupole-linear ion trap tandem mass spectrometry. *Chemical & Pharmaceutical Bulletin*.

[33] Liu J. P., Wang F., Li P. Y., Lu D. (2012). A new ocotillol-type triterpenoid saponin from red American ginseng. *Natural Product Research*.

[34] Li L., Luo G. A., Liang Q. L., Hu P., Wang Y. M. (2010). Rapid qualitative and quantitative analyses of Asian ginseng in adulterated American ginseng preparations by UPLC/Q-TOF-MS. *Journal of Pharmacy Biomedicine Analytical*.

[35] Shibata S., Tanaka O., Ando T., Sado M., Tsushima S., Ohsawa T. (1966). Chemical studies on oriental plant drugs. XIV. Protopanaxadiol, a genuine sapogenin of ginseng saponins. *Chemical & Pharmaceutical Bulletin*.

[36] Wu Q., Chen P., Zhang Q. (2016). Advances in research of chemical constituents, pharmacological activities and analytical methods of *Panax japonicus*. *Asia Pac Trad Med*.

[37] Yu J., Xu T., Lin H., Lin Y., Zhou J., Zhang Y. (2021). Comprehensive quality evaluation of American ginseng for different parts and abnormal trait based on the major ginsenoside contents and morphological characteristics. *BioMed Research International*.

[38] Wang X., Xie Q., Liu Y. (2021). Panax japonicus and chikusetsusaponins: A review of diverse biological activities and pharmacology mechanism. *Chinese Herbal Medicines*.

[39] Han S., Shi S., Zou Y. (2020). Chemical constituents from acid hydrolyzates of Panax quinquefolius total saponins and their inhibition activity to *α*-glycosidase and protein tyrosine phosphatase 1B. *Chinese Herbal Medicines*.

